# Patterns of host cell inheritance in the bacterial symbiosis of whiteflies

**DOI:** 10.1111/1744-7917.12708

**Published:** 2019-07-03

**Authors:** Xiao-Rui Xu, Na-Na Li, Xi-Yu Bao, Angela E. Douglas, Jun-Bo Luan

**Affiliations:** 1 *Liaoning Key Laboratory of Economic and Applied Entomology, College of Plant Protection, Shenyang Agricultural University, Shenyang, China;*; 2 *Department of Entomology, Cornell University, Ithaca, NY, USA and*; 3 *Department of Molecular Biology and Genetics, Cornell University, Ithaca, NY, USA*

**Keywords:** bacteriocyte, inheritance, somatic cells, symbiont transmission, whitefly

## Abstract

Whiteflies possess bacterial symbionts *Candidatus* Portiera aleyrodidium that are housed in specialized cells called bacteriocytes and are faithfully transmitted via the ovary to insect offspring. In one whitefly species studied previously, *Bemisia tabaci* MEAM1, transmission is mediated by somatic inheritance of bacteriocytes, with a single bacteriocyte transferred to each oocyte and persisting through embryogenesis to the next generation. Here, we investigate the mode of bacteriocyte transmission in two whitefly species, *B. tabaci* MED, the sister species of MEAM1, and the phylogenetically distant species *Trialeurodes vaporariorum*. Microsatellite analysis supported by microscopical studies demonstrates that *B. tabaci* MED bacteriocytes are genetically different from other somatic cells and persist through embryogenesis, as for MEAM1, but *T. vaporariorum* bacteriocytes are genetically identical to other somatic cells of the insect, likely mediated by the degradation of maternal bacteriocytes in the embryo. These two alternative modes of transmission provide a first demonstration among insect symbioses that the cellular processes underlying vertical transmission of bacterial symbionts can diversify among related host species associated with a single lineage of symbiotic bacteria.

## Introduction

Insects that live through their life cycle on nutritionally inadequate diets including vertebrate blood, plant sap and wood, derive supplementary nutrients, especially essential amino acids and B vitamins, from symbiotic microorganisms (Douglas, 1989). The microbial partners in many of these associations are intracellular bacteria localized to specialized cells that are known as bacteriocytes (Buchner, 1965). These bacteriocyte symbioses are generally evolutionarily ancient, for example, with estimated origins at least 100 million years ago for some plant sap-feeding hemipteran groups (Moran *et al*., 1993; Moran *et al*., 2005). They are sustained by reliable vertical transmission of the symbiotic bacteria from maternal bacteriocytes to the developing eggs in the ovaries (Buchner, 1965; Koga *et al*., 2012). In most associations, the bacteria are extracellular during transit to the ovaries. Exceptionally, vertical transmission of the symbionts in whiteflies (family Aleyrodidae) involves the transfer of entire bacteriocytes to the ovaries, such that one or more bacteriocytes becomes associated with each developing egg in the ovary (Buchner, 1965; Luan *et al*., 2016).

The bacterial symbiont present in all whitefly species studied is a *γ*-proteobacterium of the family Halomonadaceae, known as *Candidatus* Portiera aleyrodidium (henceforth *Portiera*) (Thao & Baumann, 2004). Recent studies of vertical transmission of bacteriocytes in the silverleaf whitefly *Bemisia tabaci* MEAM1 revealed that the bacteriocytes in adult females become mobile and move to the ovaries, where a single bacteriocyte becomes associated with the posterior pole of the terminal oocyte in each ovariole (Luan *et al*., 2016). Remarkably, this bacteriocyte persists through embryogenesis to the next generation, such that the bacteriocytes are genetically different from other cell types in the body and invariant through multiple sexual generations (Luan *et al*., 2018). These findings were obtained for a single laboratory culture and differ from the microscopical analysis of different white-fly species conducted by Paul Buchner in 1912–1918 and summarized in Buchner (1965). Although Buchner reports the transfer of bacteriocytes to ovaries to whiteflies of the genera *Trialeurodes* and *Aleorodes*, he also describes the displacement of the maternal bacteriocyte nuclei by nuclei of embryonic origin, followed by the disintegration of the maternal nuclei, in embryos of the cabbage whitefly *Aleurodes proletella*. To our knowledge, these observations have not been re-investigated with modern microscopical or genetic methods.

The principal purpose of this study was to determine whether the fates of bacteriocytes transmitted to whitefly eggs vary in different whitefly species. For this analysis, we selected *B. tabaci* MED, a member of the *B. tabaci* species complex and sister species to *B. tabaci* MEAM1, in which the bacteriocytes are inherited (Luan *et al*., 2018), and the greenhouse whitefly *Trialeurodes vaporariorum*, related to *A. prolotella*, in which maternal bacteriocytes are reportedly eliminated in the whitefly embryo (Buchner, 1965). We used multiple isolates of each species as a check for the generality of results. We investigated the genetic relationship between the bacteriocytes and other cell types, using microsatellite genotyping of the bacteriocytes and the head, which is bacteriocyte-free, and supplemented the genetic analysis with microscopical study of the fate of bacteriocytes in the embryos.

## Materials and methods

### Insects and plants

Six cultures of the whiteflies *T. vaporariorum* and *B. tabaci* MED were maintained (Tables 1 and 2), using plants grown in compost supplemented with Miracle-Gro® Water Soluble All Purpose Plant Food. The mitochondrial cytochrome oxidase I (mtCOI) gene sequence for each whitefly culture was determined by Sanger sequencing of polymerase chain reaction (PCR)-generated amplicons, using the primers and protocols of Scott *et al.* (2007) for *T. vaporariorum* and Xu *et al*. (2010) for *B. tabaci* MED. The *T. vaporariorum* culture TVC with the GenBank accession no. of MK779312 obtained from tomato (*Solanum lycopersicum*) in the greenhouse of Cornell University, USA was maintained on dwarf cherry tomato cv. Florida Lanai). The cultures TVJ and TVL with the GenBank accession nos. of MK577899 and MH422959, respectively, were collected from field populations on tobacco (*Nicotiana tabacum*) in Jilin Province and Liaoning Province, China, respectively; and they were grown on tobacco cv. NC89. Three cultures of *B. tabaci* MED, BTZ provided by Zhejiang University, BTQ provided by Qingdao Agricultural University and BTL collected from tomato in Liaoning Province, China with the GenBank accession nos. of GQ371165, MK577900 and MK500941, respectively, were reared on cotton (*Gossypium hirsutum*) cv. Shiyuan 321. All cultures were maintained in climate-controlled chambers at 26 ± 2 °C with 14 h light : 10 h dark regime.

**Table 1:** Genetic variation of head and bacteriocytes (H and B, respectively) in the whitefly *Trialeurodes vaporariorum*. The genotype of each insect is indicated by alleles scored (+), with each allele identified by its size (bp) for each microsatellite locus. Data for all microsatellite loci are provided in Data S1.

Insect	Microsatellite locus																									
	Tvap-1-1C				Tvap-2-2C						Tvap-3-1				Tvap-1-5											
	H		B		H			B			H		B		H						B					
	194	215	194	215	212	214	219	212	214	219	230	232	230	232	123	124	130	132	136	141	123	124	130	132	136	141
TVC																										
#1-20		+		+			+			+	+		+						+						+	
TVJ																										
#1		+		+	+			+			+		+				+			+			+			+
#2, 7	+	+	+	+	+			+			+	+	+	+	+					+	+					+
#3	+	+	+	+	+	+		+	+		+	+	+	+			+			+			+			+
#4	+	+	+	+			+			+	+		+		+			+			+			+		
#5	+	+	+	+	+		+	+		+	+		+		+					+	+					+
#6		+		+		+	+		+	+	+		+		+					+	+					+
#8	+	+	+	+	+	+		+	+		+	+	+	+	+					+	+					+
TVL																										
#2	+	+	+	+	+		+	+		+	+		+							+						+
#2	+		+		+			+			+	+	+	+						+						+
#3	+		+				+			+	+		+							+						+
#4	+	+	+	+			+			+	+		+			+				+		+				+
#5	+		+		+		+	+		+	+	+	+	+			+			+			+			+
#6	+		+		+			+				+		+						+						+
#7	+		+		+		+	+		+	+		+			+				+		+				+
#8	+	+	+	+	+		+	+		+	+		+					+		+				+		+

**Table 2:** Genetic variation of head and bacteriocytes (H and B, respectively) in the whitefly Bemisia tabaci MED. The genotype of each insect is indicated by alleles scored (+), with each allele identified by its size (bp) for each microsatellite locus. Data for all microsatellite loci are provided in Data S2.

Insect	Microsatellite locus																										
	WF2C01							WF2H06					WF1B11							WF1D04							
	H						B	H				B	H					B		H					B		
148	153	167	171	180	184	171	170	174	181	185	170	104	109	110	111	112	104	109	181	185	193	201	218	181	185	201
BTL																											
#1			+	+			+	+				+	+		+			+	+	+					+	+	+
#2				+			+	+				+			+			+	+	+		+			+	+	+
#3				+			+	+				+	+		+			+	+	+				+	+	+	+
#4				+			+	+				+			+			+	+	+				+	+	+	+
#5				+			+	+				+	+		+			+	+	+			+		+	+	+
#6				+			+	+				+			+			+	+				+		+	+	+
#7				+			+	+				+			+			+	+	+			+		+	+	+
#8			+	+			+	+	+			+	+		+			+	+	+			+		+	+	+
#9	+	+					+	+				+			+			+	+				+		+	+	+
#10				+			+	+				+		+		+		+	+				+		+	+	+
BTQ																											
#1,6,8,9				+			+	+				+	+					+	+	+					+	+	+
#2					+	+	+	+			+	+	+					+	+	+	+				+	+	+
#3			+		+		+	+		+		+	+					+	+	+					+	+	+
#4,10				+			+	+				+	+					+	+	+	+				+	+	+
#5					+		+	+				+	+					+	+	+					+	+	+
#7				+			+	+				+	+					+	+	+				+	+	+	+
BTZ																											
#1			+				+	+		+		+	+		+			+	+	+	+				+	+	+
#2,9				+			+	+		+		+	+		+			+	+	+					+	+	+
#3				+			+	+				+			+			+	+	+	+				+	+	+
#4				+			+	+				+	+		+			+	+	+					+	+	+
#5				+			+	+				+			+			+	+	+					+	+	+
#6			+	+			+	+	+			+	+					+	+	+					+	+	+
#7				+			+	+				+	+				+	+	+	+					+	+	+
#8				+			+	+				+	+		+			+	+	+	+				+	+	+
#10				+			+	+				+					+	+	+	+	+				+	+	+

### Genetic variation of bacteriocytes in the whitefly population

Adult females were collected haphazardly from each whitefly culture, eight insects from TVJ and TVL, 10 insects from BTL, BTQ and BTZ, and 20 insects from TVC. For each insect, the head was removed and then the bacteriocytes were dissected from the body cavity, using fresh pins and slides for each insect to avoid DNA contamination between insects. Supplementary experiments on BTZ checked the genotype of bacteriocytes over one sexual generation. Six adult female whiteflies (F0) were clip-caged individually to the abaxial surface of a leaf of cotton plants at the 6–7 true-leaf stage. After 5 days of oviposition, the F0 females were removed and dissected (as above). The eggs were reared until the F1 emerged, when one female per clip-cage was dissected.

Immediately following dissection of the insects, the DNA was extracted by the Nonidet-P40-based protocol (Delatte *et al*., 2005). Briefly, the insect material was homogenized in lysis buffer (50 mmol/L KCl, 10 mmol/L Tris-HCl pH 8.4, 0.45% Tween 20, 0.2% gelatin, 60 *μ*g proteinase K/mL and 0.45% non-ionic non-denaturing detergent IGEPAL CA-630 (Sigma-Aldrich) in USA and Nonidet P-40 (Sangon Biotech, Cat NO. A600385) in China; these detergents are functional equivalents), followed by incubation at 65 °C for 2 h and then at 100 °C for 10 min to inactivate the proteinase K. Finally, samples were stored at −20 °C.

The microsatellite profile of each sample was determined for four published microsatellite loci developed for *B. tabaci* (Hadjistylli *et al*., 2014) and *T. vaporariorum* (Ovcarenko *et al*., 2014). The PCR reactions (10 *μ*L) comprised 60 ng gDNA, 5 *μ*L2× Multiplex PCR Master Mix (Qiagen, Hilden, Germany) and 0.2 *μ*mol/L fluorescent labeled primers (Table S1). The cycling schedule was: 5 min at 95 °C, 35 cycles with 95 °C for 30 s, 60 °C for 1 min 30 s, 72 °C for 30 s and final extension cycle of 68 °C for 30 min. Diluted PCR products (1 : 2 with nuclease-free water) were mixed with HiDi formamide (Thermo Fisher Scientific, Waltham, MA, USA) and LIZ 500 ladder (Applied Biosystems, Foster City, CA, USA) and were analyzed on a capillary sequencer, ABI 3730xl DNA Analyzers (Applied Biosystems).

The microsatellite profiles were analyzed using the software Genemarker (SoftGenetics LLC., USA) following the user manual. In parallel, the PCR products from bacteriocytes of the whitefly *B. tabaci* MED were Sanger sequenced, and the sequence identity was determined by Basic Local Alignment Search Tool to the genomes of the whitefly *B. tabaci* MED (National Center for Biotechnology Information [NCBI] accession numbers: GCA 003994315.1) and *Portiera* (NCBI accession numbers: GCA 000827855.1) in the NCBI database. The allelic richness for the four microsatellite markers across each whitefly population was calculated using the software Fstat294 (https://www2.unil.ch/popgen/softwares/fstat.htm), and genetic diversity indices and significant departure from Hardy-Weinberg equilibrium at each locus was tested in GENEPOP (http://genepop.curtin.edu.au/) with the exact probability test (Rousset, 2008). Further, the genotypes for single and all four microsatellite loci across three populations of the whitefly *T. vaporariorum* and *B. tabaci* MED, respectively, were compared. Microsatellite loci in heads and bacteriocytes of the whitefly *B. tabaci* MED in this study was also compared to the whitefly *B. tabaci* MEAM1 (Luan *et al*., 2018).

### Microscopical analysis of bacteriocyte dynamics during whitefly oogenesis and embryogenesis

For *T. vaporariorum*, the experimental material comprised ovaries and eggs derived from 30 female adults of culture TVC that had been allowed to lay eggs for 1 h on tomato plants. The ovaries were dissected from the insects using fine pins, and then fixed in 4% paraformaldehyde (PFA) in cytoskeleton buffer (10 mmol/L MES pH 6.1, 150 mmol/L NaCl, 5 mmol/L ethylene glycol tetraacetic acid, 5 mmol/L glucose, and 5 mmol/L MgCl_2_) at room temperature for 1 h, and permeabilized with 0.1% Triton X-100 in phosphate-buffered saline (PBS) for 30 min. Following washing in PBS, the samples were incubated with Hoechst 33342 (10 *μ*g/mL PBS, Thermo Scientific) overnight at 4 °C. Eggs were collected at day-1 after deposition, punctured using a pin and processed exactly as in Luan *et al*. (2018). Briefly, the punctured eggs were fixed in 4% PFA at 4 °C overnight, permeabilized with 0.1% Triton X-100 in PBS at room temperature for 2 h, and incubated with Hoechst 33342 in PBS overnight at 4 °C. All images were collected and analyzed on a Zeiss LSM 700 confocal microscope.

Supplementary experiments monitored the number of bacteriocytes associated with oocytes in dissected ovaries, deposited eggs and newly hatched nymphs. We analyzed z-stacks obtained by optical sectioning of live material to determine the number of the bacteriocytes in the ovarioles and eggs at day-1 post-oviposition for *T. vaporariorum* TVL on tobacco and *B. tabaci* MED BTZ on cotton, with 14 replicates per time point. The number of bacteriocytes in late embryos of *B. tabaci* MED was determined by dissection. This method yielded unreliable results for *T. vaporariorum* embryos, and the analysis of this species was, therefore, conducted by dissections of newly hatched nymphs.

## Results

### Microsatellite analysis

Overall, *T. vaporariorium* was polymorphic for all four microsatellite loci tested, with 2–6 alleles per locus (Table 1; Data S1). However, the genetic variation differed between the three populations tested. All 20 individuals of TVC were homozygous for the same allele for each locus, but each of the TVJ and TVL insects had a unique microsatellite profile, apart from two individuals of TVJ (#2, #7), with allele frequencies for all loci conforming to the Hardy-Weinberg distribution (Table S2a). For every insect, the microsatellite profile of the bacteriocytes and head were identical.

The microsatellite profiles of *B. tabaci* MED differed from *T. vaporariorum* in that the microsatellite profile of the *B. tabaci* bacteriocytes was uniform across all the insects tested and different from the head profile in every insect (Table 2; Data S2). For the heads, 4–6 alleles of each of the four microsatellite loci were scored, all were polymorphic apart from WF1B11 (with allele-104 fixed in BTQ), and most were in Hardy-Weinberg equilibrium (Table S2b). The bacteriocyte samples yielded one allele for the loci WF2C01 and WF2H06, two alleles for WF1B11, and three alleles for WF1D04. Two of the alleles in the bacteriocytes were present in all or most of the heads (WF2H06 and WF2C01, respectively), but several fixed alleles in bacteriocytes were rare in the head samples, for example 109 bp allele of WF1B11, detected in the head of just one insect (BTL10) (Table 2; Data S2). Sanger sequencing of the microsatellite alleles recovered from bacteriocytes yielded 93%–100% sequence identity with the published *B. tabaci* MED genome sequence, and no sequence similarity to the genome of the bacterial symbiont *Portiera*. A supplementary analysis tested for the effect of sexual reproduction on the genotype of the bacteriocytes in *B. tabaci* MED. Using culture BTZ, six crosses were set up, and the microsatellite profile of the head and bacteriocytes of the first (F0) and second (F1) generation of females was determined. The alleles scored for the heads differed between F0 and F1 insects for two or three of the six crosses at each locus, but the microsatellite profile for the bacteriocytes was invariant and included one allele (#109 for locus WF1B11) that was not represented in any of the insects in this experiment (Table 3; Data S2).

**Table 3:** Genetic variation of bacteriocytes over one sexual generation of the whitefly *Bemisia tabaci* MED culture BTZ. Six adult females (generation F0) were mated and allowed to deposit eggs (generation F1), and then the microsatellite profile of dissected bacteriocytes (B) and head (H) were scored. The same microsatellite analysis was conducted on one female offspring of each cross (F1). The genotype of each insect is indicated by alleles scored (+). Data for all microsatellite loci are provided in Data S2.

Insect	Microsatellite WF2C01			Microsatellite WF2H06			Microsatellite WF1B11					Microsatellite WF1D04					
	H		B	H		B	H			B		H			B		
	167	171	171	170	174	170	104	110	112	104	109	181	185	201	181	185	201
Insect #1																	
F0		+	+	+		+		+		+	+	+	+		+	+	+
F1	+	+	+	+		+		+		+	+	+	+		+	+	+
Insects #2 and #5																	
F0		+	+	+		+		+		+	+	+	+		+	+	+
F1		+	+	+	+	+	+	+		+	+	+			+	+	+
Insect #3																	
F0		+	+	+	+	+	+	+		+	+	+	+		+	+	+
F1		+	+	+		+	+	+		+	+	+	+		+	+	+
Insect #4
F0		+	+	+		+		+		+	+	+		+	+	+	+
F1	+	+	+	+		+		+	+	+	+	+	+		+	+	+
Insect #6																	
F0		+	+	+		+			+	+	+	+	+		+	+	+
F1		+	+	+		+			+	+	+	+	+		+	+	+

### Microscopical analysis of bacteriocytes in ovarioles and eggs of the whitefly

**Fig. 1:**
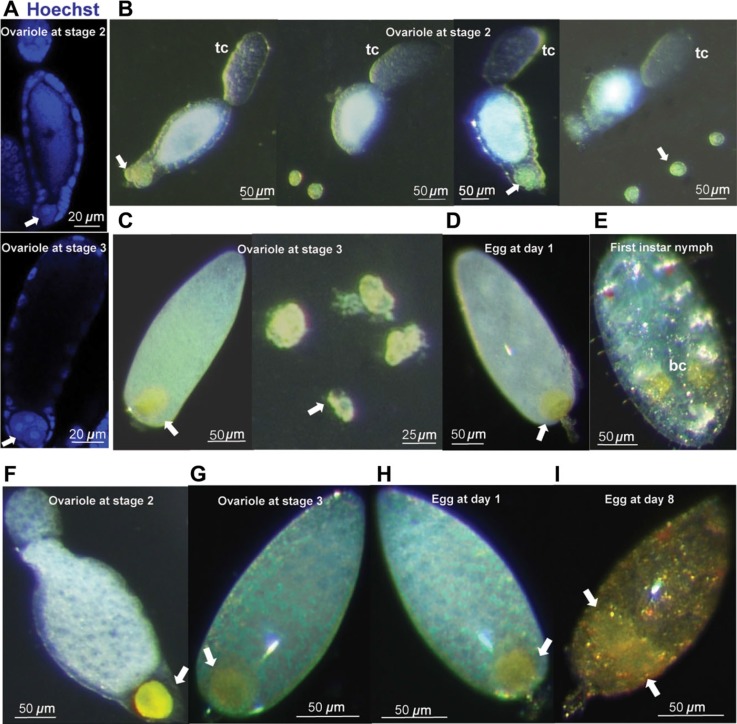
Vertical transmission of bacteriocytes in the whitefly *Trialeurodes vaporariorum* and *Bemisia tabaci* MED. (A) Localization of bacteriocytes and associated nuclei in ovarioles at stages 2 and 3 in the whitefly *T. vaporariorum*, revealed by Hoechst 33342 staining of DNA (blue) and (B–E) localization of bacteriocytes (denoted by the white arrow) in ovarioles at stage 2 (B) and stage 3 (C), the egg at day-1 post-oviposition (D) and in the 1st instar nymph (E) in the whitefly *T. vaporariorum*, revealed by light microscopy. The bacterial symbionts that pack the cytoplasm of bacteriocytes are evident in the bacteriocyte periphery. The dissected bacteriocytes at the right side were placed together with the ovarioles at the left side. tc, trophocytes. (F–I) Localization of bacteriocytes (denoted by the white arrow) in ovarioles at stage 2 (F) and stage 3 (G) and in the egg at 1 day (H) and 8 days (I) post-oviposition of the whitefly *B. tabaci* MED.

To investigate the mode of bacteriocyte inheritance in the two whitefly species, we analyzed the bacteriocyte dynamics during oogenesis and embryogenesis. Consistently, a single mature oocyte of *T. vaporariorum* was colonized by multiple bacteriocytes, while a single bacteriocyte was associated with each oocyte of *B. tabaci* MED (Fig. 1).

Subsequent studies determined the timing of bacteriocyte colonization of the oocytes. Bacteriocytes were not detected in association with immature oocytes, which are small and associated with large trophocytes. In *T. vaporariorum*, one to three bacteriocytes were found with large oocytes (associated with small trophocytes); and fully mature oocytes (lacking trophocytes) invariably bore four bacteriocytes (Fig. 1A–C). Four bacteriocytes were also scored in each in eggs at day-1 after deposition, and newly hatched larvae bore 10 ± 0.4 (mean ± SE, 14 replicates) bacteriocytes (Fig. 1D, E). In contrast, the bacteriocyte counts associated with oocytes of *B. tabaci* MED matched the published data for *B. tabaci* MEAM1 (Luan *et al*., 2016; Luan *et al*., 2018), with a single bacteriocyte per oocyte that divides once just prior to egg hatching (Fig. 1F–I).

## Discussion

The key result of this study is the genetic evidence that the fate of bacteriocytes transferred to oocytes differs between the whitefly species *B. tabaci* MED and *T. vaporariorum*. Specifically, the identical microsatellite profiles in heads and bacteriocytes of *T. vaporariorum* is fully consistent with the conclusion based on microscopical analysis (Buchner, 1965) that bacteriocytes in the related species *A. proletella* are of embryonic origin; while the demonstration that the microsatellite profiles of bacteriocytes are consistently different from the profile of heads in *B. tabaci* MED demonstrates that this mode of inheritance is not special to the single laboratory culture of *B. tabaci* MEAM1 studied by Luan *et al*. (2018). Our microscopical analysis revealed an additional difference between the two species in bacteriocyte interactions with the whitefly eggs. We found that multiple bacteriocytes are transferred to *T. vaporariorum* oocytes, as previously reported for *A. proletella* (Buchner, 1965), but a single bacteriocyte is transferred to each *B. tabaci* MED oocyte, as for *B. tabaci* MEAM1 (Luan *et al*., 2018). However, we were unable to investigate the timing of dissolution of the maternal bacteriocyte nuclei in the *T. vaporariorum* embryos by microscopical methods because of difficul-ties in visualizing bacteriocyte nuclei in the late embryos of this species.

The genetic evidence for two alternative fates of bacteriocytes transmitted to whitefly oocytes raises the linked evolutionary issues of the likely ancestral condition and the number and direction of transitions between degradation and persistence of maternal bacteriocytes in whitefly embryos. These questions can, in principle, be addressed by a phylogenetically informed investigation of the fate of maternal bacteriocytes in whitefly species representative of the full taxonomic diversity of this insect group. However, from basic biological principles, the persistent bacteriocyte phenotype is predicted to be evolutionarily short-lived, and consequently the derived state. This is because these somatic cells are asexual with no known capacity to eliminate deleterious mutants by recombination (Muller, 1932; Felsenstein, 1974), although the possibility cannot be excluded that their evolutionary lifespan may be extended by unconventional and poorly understood processes for genetic exchange (Mirzaghaderi & Horandl, 2016; Wilson *et al*., 2018).

These considerations lead to the expectation that, ancestrally, the symbiosis was transmitted via bacteriocytes that degrade in the whitefly embryo. The persistent bacteriocytes may have evolved in *B. tabaci* by a lesion in the developmental program orchestrating the death of maternal bacteriocyte nuclei. Alternatively, the ancestral bacteriocyte may have been replaced by a different cell lineage that is capable of accommodating the bacterial symbionts and had immortalized properties. Similarly, the predicted genomic deterioration of the persistent bacteriocyte lineage could lead to the evolutionary replacement by a non-persistent cell lineage. This reasoning leads to the possibility of repeated evolutionary transitions between persistent and non-persistent bacteriocytes across the whitefly phylogeny, possibly involving multiple cell lineages of different developmental origins. Immediately relevant to these general issues is the evolutionary relationship between the persistent bacteriocytes in *B. tabaci* MED, studied here, and *B. tabaci* MEAM1, investigated by Luan *et al*. (2018). MED and MEAM1 are sister species, with divergence time estimated, variously, as 0.63–2.88 mya (Santos-Garcia *et al*., 2015), 5–6 mya (Mugerwa *et al*., 2018), and 13 mya (Boykin *et al*., 2013). Additional genetic data will facilitate testing whether the bacteriocytes in the two species are derived from the same lineage of persistent bacteriocytes present in their common ancestor, are independently evolved in the two species, or have even arisen multiple times within a single species. However, the presence of bacteriocyte microsatellite alleles that are absent from the heads of the *B. tabaci* MED cultures studied here suggests that the persistent bacteriocytes have likely not very recently evolved.

We conclude by considering the general relevance of our findings to understanding of the vertical transmission mechanisms in insect symbioses (Koga *et al*., 2012). The spatio-temporal organization of transmission varies widely among different insect associations, with respect to developmental timing and pattern of bacteriocyte-ovary interaction (Douglas, 1989; Bright & Bulgheresi, 2010). Despite this variation, recent studies are indicative of common molecular mechanisms, including a role of homeodomain transcription factors, in different systems (Braendle *et al*., 2003; Matsuura *et al*., 2015). However, here is a general expectation in the literature of molecular and cellular uniformity of transmission mechanisms among related insects bearing microbial symbionts of the same lineage (Buchner, 1965; Bright & Bulgheresi, 2010). Our research on whitefly symbiosis provides the contrary evidence for evolutionary diversification of transmission mechanism within a single insect group.
