# Supplementary material for: Patterns of host cell inheritance in the bacterial symbiosis of whiteflies
Source: Insect Sci. 2019 Jul 3:10.1111/1744-7917.12708. doi: 10.1111/1744-7917.12708 (PMC7198116; doi:10.1111/1744-7917.12708)
Supplement: Supplementary file 1 [file IS-2019-1744-7917-12708-s1.docx]

Table S2. Genetic diversity indices for microsatellite loci (NA, no information; ns, not significant)

| Culture | Locus | | | No. of alleles  Na | | Allelic richness  A_R_ | | Fit to Hardy-Weinberg (HW) distribution | | | | | | |
| --- | --- | --- | --- | --- | --- | --- | --- | --- | --- | --- | --- | --- | --- | --- |
|  |  |  |  |  |  |  |  | Observed heterozygosity  H_O_ | | Expected heterozygosity  H_E_ | | Inbreeding coefficient  *F_IS_* | | Significance of departure from HW^1^ |
| 1. *Trialeuroides vaporariorum* | | | | | | | | | | | | | | |
| TVC | Tvap-1-1C | | | 1 | | 1 | | 0 | | 0 | | NA | | NA |
| N=20 | Tvap-2-2C | | | 1 | | 1 | | 0 | | 0 | | NA | | NA |
|  | Tvap-3-1 | | | 1 | | 1 | | 0 | | 0 | | NA | | NA |
|  | Tvap-1-5 | | | 1 | | 1 | | 0 | | 0 | | NA | | NA |
| TVJ | Tvap-1-1C | | | 2 | | 2 | | 6 | | 4 | | -0.56 | | ns |
| N=8 | Tvap-2-2C | | | 5 | | 3 | | 4 | | 5 | | 0.21 | | ns |
|  | Tvap-3-1 | | | 2 | | 2 | | 4 | | 3.2 | | -0.27 | | ns |
|  | Tvap-1-5 | | | 3 | | 4 | | 8 | | 5.53 | | -0.49 | | ns |
| Over all loci (mean±S.E.) | | | | 3±0.7 | | 2.8±0.5 | | 5.5±0.95 | | 4.43±0.52 | | -0.28±0.17 | | - |
| TVL | Tvap-1-1C | | | 2 | | 2 | | 3 | | 2.6 | | -0.17 | | ns |
| N=8 | Tvap-2-2C | | | 3 | | 2 | | 4 | | 4.27 | | 0.07 | | ns |
|  | Tvap-3-1 | | | 3 | | 2 | | 2 | | 3.2 | | 0.39 | | ns |
|  | Tvap-1-5 | | | 4 | | 4 | | 4 | | 3.53 | | -0.14 | | ns |
| Over all loci (mean±S.E.) | | | | 3±0.4 | | 2.5±0.5 | | 3.25±0.48 | | 3.4±0.35 | | 0.04±0.13 | | - |
| Over all sites and loci | | | | 2.3±0.4 | | 2.1±0.3 | | 2.92±0.75 | | 2.61±0.6 | | -0.12±0.12 | | - |
| (mean±S.E.) | | | |  | |  | |  | |  | |  | |  |
| 1. *Bemisia tabaci* MED | | | | | | | | | | | | | | |
| Culture | | Locus | No. of alleles  Na | | Allelic richness  A_R_ | | Fit to Hardy-Weinberg (HW) distribution | | | | | | | |
|  |  |  |  |  |  |  | Observed heterozygosity  H_O_ | | Expected heterozygosity  H_E_ | | Inbreeding coefficient  *F_IS_* | | Significance of departure from HW^1^ | |
| BTL | | WF2C01 | 3 | | 4 | | 3 | | 3.63 | | 0.18 | | ns | |
| N=10 | | WF2H06 | 2 | | 2 | | 1 | | 1 | | NA | | NA | |
|  | | WF1B11 | 3 | | 4 | | 5 | | 4.9 | | -0.02 | | ns | |
|  | | WF1D04 | 5 | | 4 | | 6 | | 6.58 | | 0.09 | | ns | |
| Over all loci (mean±S.E.) | | | 3.3±0.6 | | 3.5±0.5 | | 3.75±1.11 | | 4.03±1.18 | | 0.08±0.06 | | - | |
| BTQ | | WF2C01 | 4 | | 4 | | 2 | | 4.9 | | 0.60 | | ** | |
| N=10 | | WF2H06 | 3 | | 3 | | 2 | | 1.95 | | -0.03 | | ns | |
|  | | WF1B11 | 1 | | 1 | | 0 | | 0 | | NA | | NA | |
|  | | WF1D04 | 3 | | 3 | | 4 | | 3.53 | | -0.14 | | ns | |
| Over all loci (mean±S.E.) | | | 2.8±0.6 | | 2.8±0.6 | | 2±0.82 | | 2.59±1.05 | | 0.14±0.23 | |  | |
| BTZ | | WF2C01 | 3 | | 2 | | 1 | | 2.68 | | 0.64 | | ns | |
| N=10 | | WF2H06 | 3 | | 3 | | 4 | | 3.53 | | -0.14 | | ns | |
|  | | WF1B11 | 5 | | 3 | | 6 | | 6.47 | | 0.08 | | ns | |
|  | | WF1D04 | 2 | | 2 | | 4 | | 3.37 | | -0.20 | | ns | |
| Over all loci (mean±S.E.) | | | 3.3±0.6 | | 2.5±0.3 | | 3.75±1.03 | | 4.01±0.84 | | 0.09±0.19 | | - | |
| Over all sites and loci | | | 3.1±0.3 | | 2.9±0.3 | | 3.17±0.58 | | 3.54±0.58 | | 0.11±0.09 | | - | |
| (mean±S.E.) | | |  | |  | |  | |  | |  | |  | |
